# Impression Material Mass Retained in the Mucobuccal Fold

**DOI:** 10.1155/2014/416965

**Published:** 2014-06-29

**Authors:** N. Karam Genno, A. Assaf

**Affiliations:** ^1^Department of Oral and Maxillofacial Radiology, Dental School, Lebanese University, Beirut, Lebanon; ^2^Beirut Arab University, Beirut, Lebanon; ^3^Department of Prosthodontics, Lebanese University, Beirut, Lebanon

## Abstract

Trapped foreign bodies and tissue reactions to foreign materials are commonly encountered in the oral cavity. Traumatically introduced dental materials, instruments, or needles are the most common materials referred to in the dental literature. This paper describes an iatrogenic foreign body encapsulation in the oral mucosa, clinically appearing as 5 × 10 mm tumor-like swelling with an intact overlying epithelium and diagnosed as a polymeric impression material. Detailed case history and, clinical and radiographic examinations including CBCT and spectrometric analysis of the retrieved sample were necessary to determine accurately the nature, size, and location of the foreign body. It is suggested that the origin of the material relates to an impression made 2 years ago, leaving a mass trapped in a traumatized mucosal tissue.

## 1. Introduction

Foreign bodies implanted in the region of the oral cavity are described periodically in the dental literature, but recent reports are rare. This could be due to the more common use of rubber dams and techniques that avoid trauma and implantation in tissues. Often the foreign bodies reported are dental materials, metallic projectiles, and glass. While most documented cases present patients with oral pain and signs of inflammation with purulent discharge, asymptomatic foreign bodies affecting the oral cavity are rarely reported in the literature [1].

This paper describes a case with an asymptomatic lesion presenting as a benign tumor-like mass, which was later found to be rubber-based impression material implanted in the mucobuccal fold.

## 2. Case History

A 29-year-old woman attended the operatory with a discomfort between teeth number 26 and 27. The patient's past dental history included multiple restorative procedures. There was nothing significant in her medical history.

**Figure 1:**
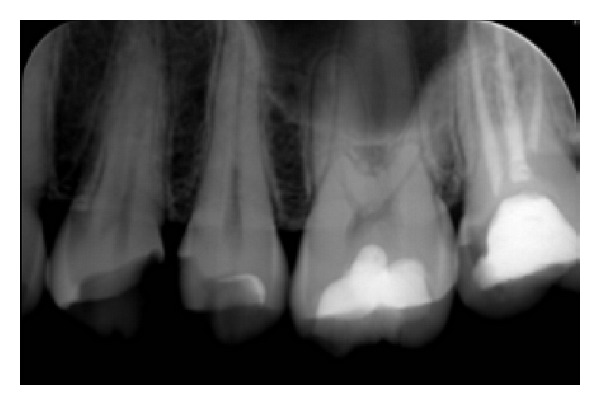
Initial radiography showing proximal cavity on tooth number 27.

On the clinical examination of the oral cavity, food retention between 26 and 27 was detected with a recurrent decay under the composite filling material of the tooth number 27. The radiography of this tooth revealed a root canal treatment. The mesial and palatal extension of the decay justified the indication of a crown (Figure 1).

**Figure 2:**
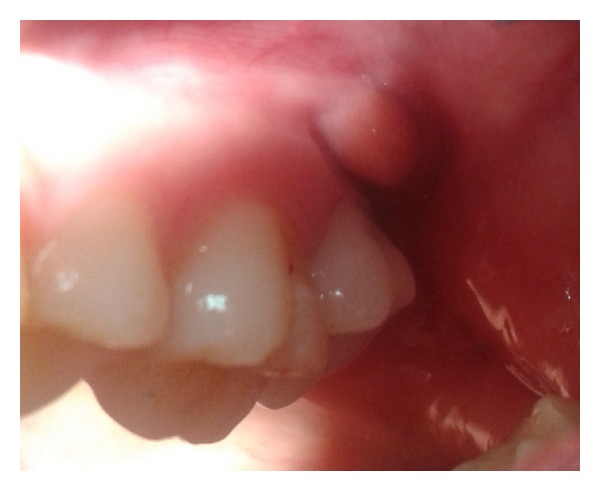
Oral examination revealed a cyst-like swelling in the depth of the vestibule of tooth number 27.

Fortuitously, a submucosal mass was simultaneously identified in the same region. When stretching the cheek, a 5 × 10 mm nodule was visible in the mucobuccal fold region. The mass was soft to rubbery-firm on palpation, with no surrounding indurations. It was minimally compressible and margins were well defined. The patient experienced no pain on palpation and was unaware of the presence and duration of the lesion (Figure 2).

A CBCT was taken to define the entire extent and nature of the lesion and its relationship with the surrounding structures (Figures 4(a), 4(b), and 4(c)).

**Figure 3:**
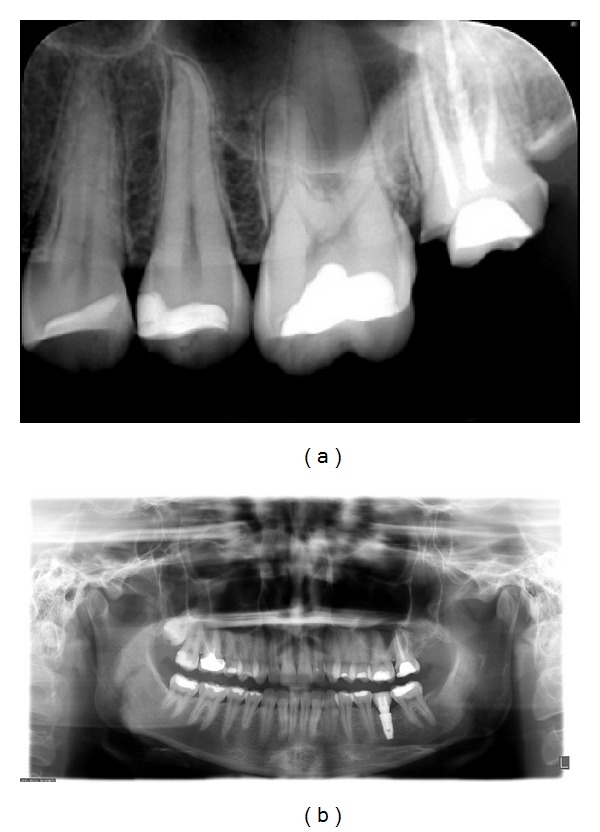
(a) and (b) Periapical and panoramic films show no evidence of lesion in the upper region of left second molar.

**Figure 4:**
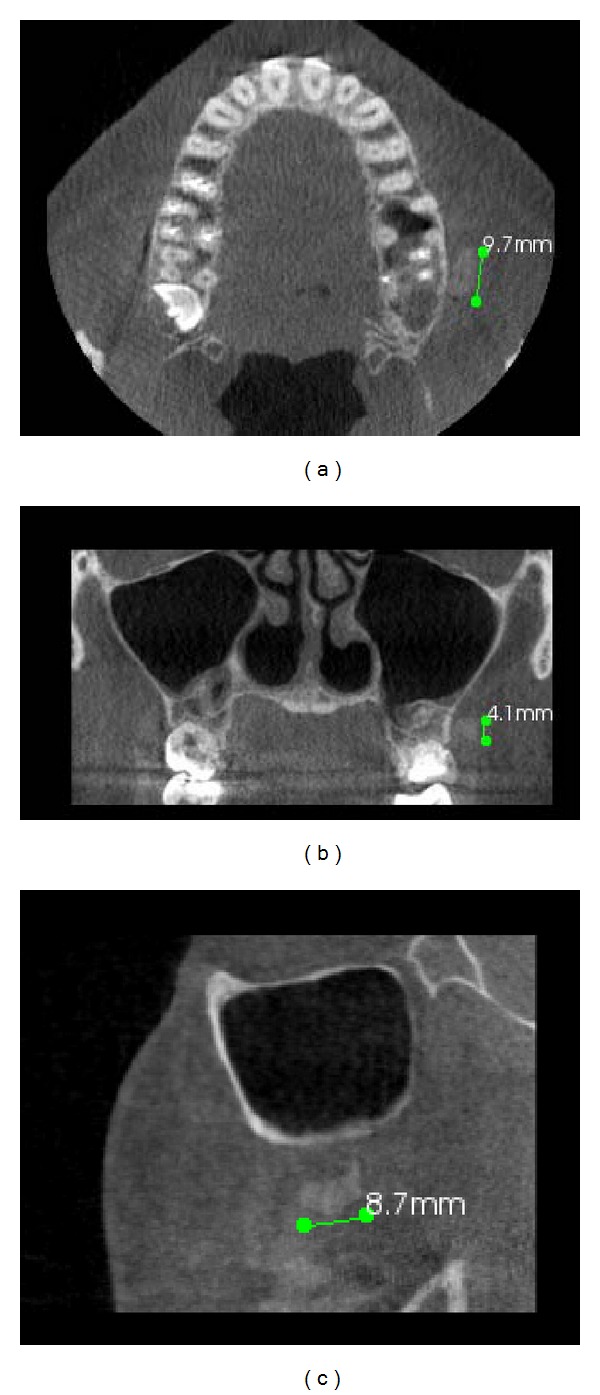
(a) CBCT axial cuts showing the extent of the radio-opacity of the lesion in the vestibular side and then in relationship with the second molar. (b) and (c) Coronal and sagittal CBCT scan image demonstrating also the radio-opacity at the same level of the coronal 1/3 of the root canal. The lesion measured approximately 9.7 × 8.7 × 4.1 mm.

The lesion was not identified on periapical and panoramic radiographs (Figures 3(a) and 3(b)).

The patient consented for a biopsy for a definitive diagnosis of the mass and immediate removal if needed. Excision biopsy was performed under local anesthesia. The mass was bluntly dissected and removed in total and placed in 10% formalin for routine histopathologic examination.

**Figure 5:**
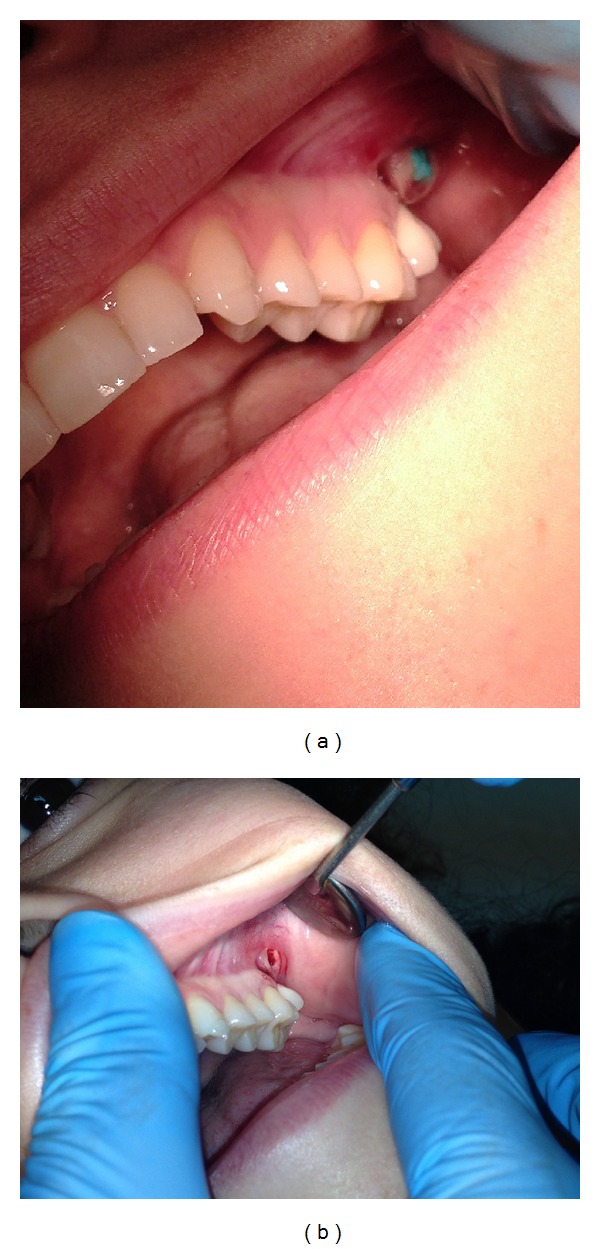
(a) and (b) Bluish mass found in the mucosal tissue and removed by excision.

On gross examination, the mass was bluish in color and there was evidence of a thin overlying fibrous capsule (Figures 5(a) and 5(b)).

The result of the microscopic examination revealed an acellular lamellar and pigmented mass with no signs of cellular tissue or associated inflammation.

The Fourier transform infrared (FTIR) spectroscopic analysis for identification of the sample by comparing it to three other elastomeric impression materials showed similar infrared spectrum of absorptions. The results strongly suggest a chemistry of a polyvinyl siloxane polymer for the retrieved mass (Figure 7).

The differential diagnosis of the mass included lipoma, granulation tissue, fibroma, and foreign body granuloma. Lipoma was considered most likely because of the morphology and consistency on clinical palpation and absence of symptoms [1].

The final diagnosis was intramucosal foreign body consistent with rubber-based impression material. Upon further questioning and discussion with the patient's previous dentist, it was confirmed that the patient had previous amalgam restorations removed in the right region at the first upper molar and subsequent crown placement. Impression material used for fabrication was polyvinyl siloxane.

## 3. Discussion

Foreign bodies may be deposited in the oral cavity either by traumatic injury or iatrogenically [2]. Tissue reactions are therefore expected. The more common lesions include restorative materials, endodontic obturation materials deposited apically, mucosal amalgam and graphite tattoos, myospherulosis, oil granulomas, and traumatically introduced dental materials and instruments [3].

The use of elastomeric impression materials including polysulfide rubber base, polyether, reversible hydrocolloid, and vinyl polysiloxane silicone rubber base in fixed prosthodontic procedures is routine and usually without adverse consequences. However, reports in the literature have indicated problems of pain and swelling after its use, allergic response, localized inflammation and bone loss, and foreign body response to retained impression material [4].

Dental impression materials are manufactured to be biocompatible and have minimal cytotoxic effects. Studies have shown that there is a low probability of allergic or toxic reactions. Cytotoxic tests on cell cultures have shown that polyethers are more toxic than vinyl polysiloxanes [5, 6]. Concomitantly, for cytotoxicity evaluation, a contact time of 15 or 30 min between cell and dental impression materials is needed to be more reflective of clinical situations, knowing that significant (*P* < 0.05) differences of cell viability and cell proliferation have been found between the “polymerizing” and “set” impression materials. Also, significant (*P* < 0.05) differences were noted with variance in duration of time [7].

All reported cases of impression materials presenting as a foreign body embedded in the soft tissues describe an associated inflammatory reaction which is a response of the biological tissue to the foreign material [8]. The foreign body reaction consists of protein adsorption, macrophages, multinucleated foreign body giant cells (macrophage fusion), fibroblasts, and angiogenesis [9].

Impression materials' radiodensity is influenced by composition. Different materials and respective classes had a different behavior with respect to radiodensity. Polysulfides showed high values of radiodensity, comparable to human enamel (*P* > 0.05) but not to bovine enamel (*P* < 0.05). Human dentin was similar only to a heavy-body addition silicon material, but bovine dentin was similar to several materials. Generally, heavy-body materials showed higher radiodensity than light-body ones (*P* < 0.05) [10].

**Figure 6:**
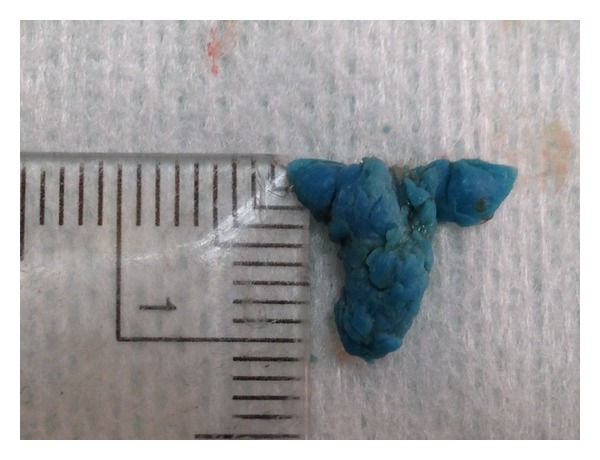
Measurements of the excised mass revealed dimensions of 10 × 7.5 mm.

**Figure 7:**
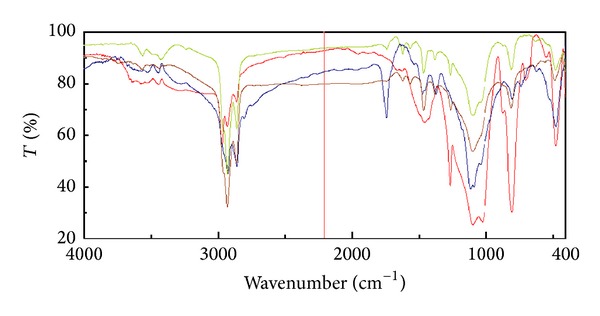
Results from infrared absorption spectroscopy, using the Fourier transform infrared (FTIR) spectrometer.

The biocompatible nature of the material may explain the pseudoencapsulation around the excised lesion in our patient as the body's mechanism to wall off the foreign body. Radiographs aid in the location of foreign bodies if the material is dense enough to appear radiopaque. If the material is radiolucent, it may be identified if it is present in considerable thickness or density [1]. The reduced thickness of the mass at edge regions explains why the direct measurements are greater than the measurements made on the image (Figure 6).

We can therefore suggest that the foreign body implanted within the mucosa may be the result of a material being forced through the tissue that has been traumatized during impression making following crown preparation in the contralateral side. Indeed, the upper right molar has been restored around 2 years ago with a porcelain-fused-to-metal crown.

## 4. Conclusion

The use of elastomeric impression materials is routine in restorative procedures. Special care must be taken to avoid leaving fragments of the material in the tissues as adverse soft tissue responses might occur, whether symptomatic or not. Clinicians should be aware that intramucosal foreign body can be an incidental finding on intraoral examinations and can mimic the appearance of a benign and well-defined connective tissue tumor. However, real-size dimensions might be larger than what CBCT image might show. It is therefore advised that after making the impression, all tissues which involve exposed bone or traumatized soft tissue must be well irrigated to remove any left residue.
